# Reviewed article: Müller CH, Bertoldi AD, Bielemann RM, Machado KP, Tomasi E, Gonzalez MC, Silveira MPT. Prevalence of polypharmacy use and association with mortality: a cohort study of elderly people in Southern Brazil, 2014-2017. Epidemiol Serv Saude. 2025;34:e20240081. 10.1590/S2237-96222025v33e20240081.en

**DOI:** 10.1590/S2237-96222024v33e20240081.a

**Published:** 2025-04-07

**Authors:** Aluísio Oliveira

**Affiliations:** 1 Instituto Federal de Educaçõa Ciência e Tecnologia do Sudeste de Minas Gerais, Juiz de Fora, Minas Gerais, Brasil

## Peer review A

## Questionnaire

Does the manuscript contain new and significant information to justify publication? Yes

Does the Abstract (Summary) clearly and accurately describe the content of the article? Yes

Is the problem significant and concisely stated? Yes

Are the methods described comprehensively? Yes

Are the interpretations and conclusions justified by the results? Yes

Is adequate reference made to other work in the field? Yes

## Manuscript Structure

Length of article is: Adequate

Number of tables is: Adequate

Number of figures is: Adequate

Please state any conflict(s) of interest that you have in relation to the review of this paper (state “none” if this is not applicable).

None

Interest: Excellent

Quality: Excellent

Originality: Excellent

Overall: Excellent

Recommendation: Accept

## Comments

Necessidade de ajustes pontuais no texto.

